# Supplementary material for: Evaluation of subclinical ventricular systolic dysfunction assessed using global longitudinal strain in liver cirrhosis: A systematic review, meta-analysis, and meta-regression
Source: PLoS One. 2022 Jun 7;17(6):e0269691. doi: 10.1371/journal.pone.0269691 (PMC9173645; doi:10.1371/journal.pone.0269691)
Supplement: S7 Fig — SD , standard deviation; IV , inverse variance; CI , confidence interval; df , degrees of freedom; Chi2 , chi-squared statistic; p , p-value; I2 , I-squared heterogeneity statistic; Z , Z statistic. (DOCX) [file pone.0269691.s008.docx]

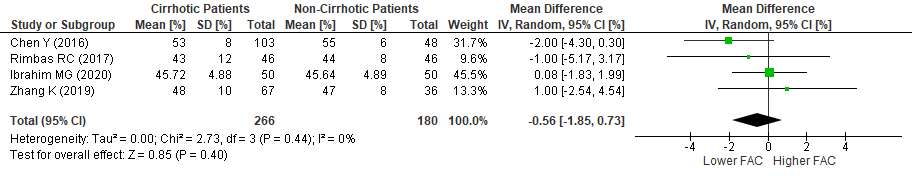


**S7 Fig.** The mean difference in right ventricular fractional area change between patients with and withour cirrhosis. SD , standard deviation; IV ,  inverse variance; CI ,  confidence interval; df ,  degrees of freedom; Chi^2^ , chi-squared statistic; p ,   p-value; I^2^ ,  I-squared heterogeneity statistic; Z , Z statistic
