# Supplementary figures and images for: Evaluation of subclinical ventricular systolic dysfunction assessed using global longitudinal strain in liver cirrhosis: A systematic review, meta-analysis, and meta-regression
Source: PLoS One. 2022 Jun 7;17(6):e0269691. doi: 10.1371/journal.pone.0269691 (PMC9173645; doi:10.1371/journal.pone.0269691)

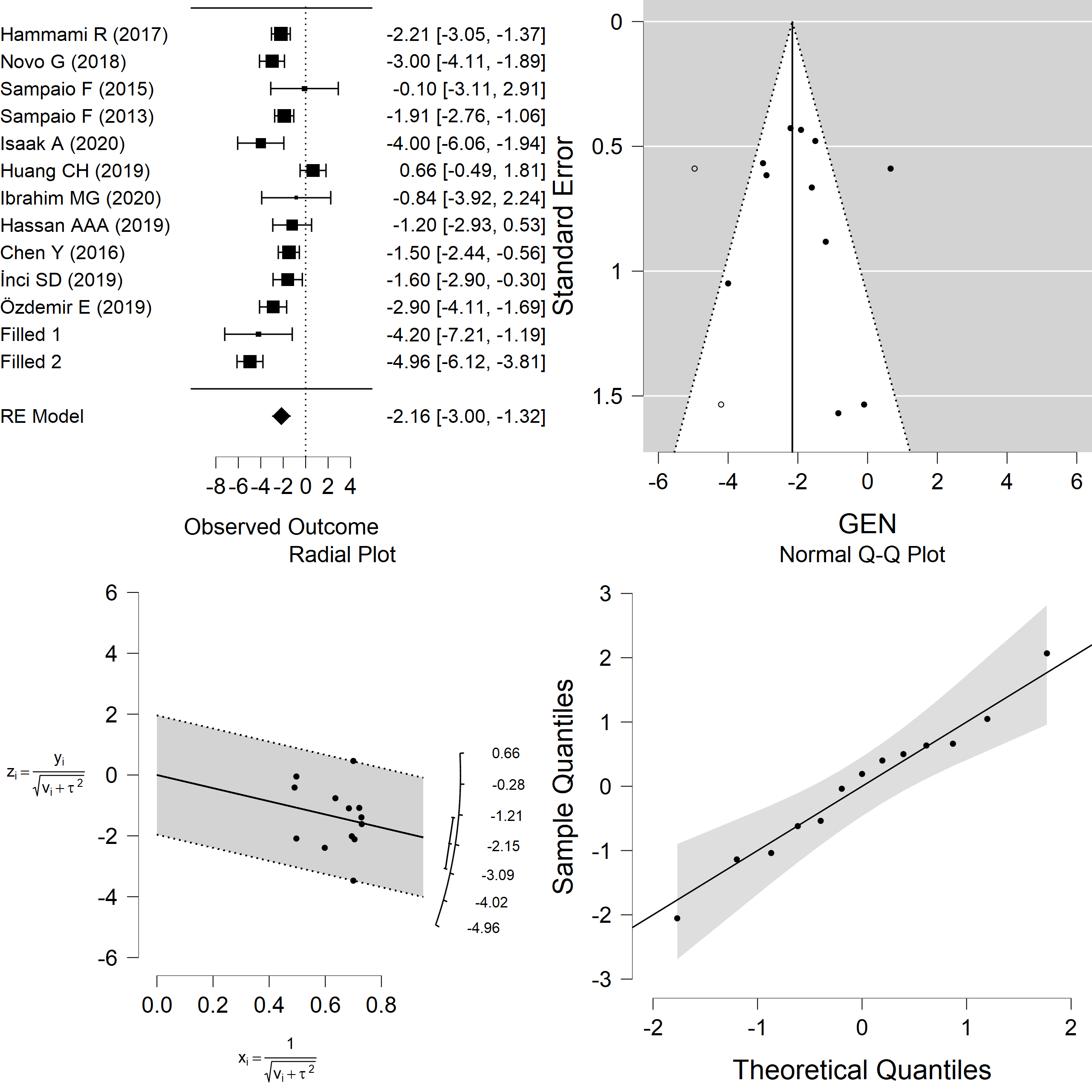


**S9 Fig.** Trim-and-fill analysis for the funnel plot in Fig 5. GEN generated the mean difference.

Supplement: S9 Fig — GEN generated the mean difference. (DOCX) [file pone.0269691.s010.docx]
