# Supplementary material for: Evaluation of subclinical ventricular systolic dysfunction assessed using global longitudinal strain in liver cirrhosis: A systematic review, meta-analysis, and meta-regression
Source: PLoS One. 2022 Jun 7;17(6):e0269691. doi: 10.1371/journal.pone.0269691 (PMC9173645; doi:10.1371/journal.pone.0269691)
Supplement: S13 Fig — (DOCX) [file pone.0269691.s014.docx]

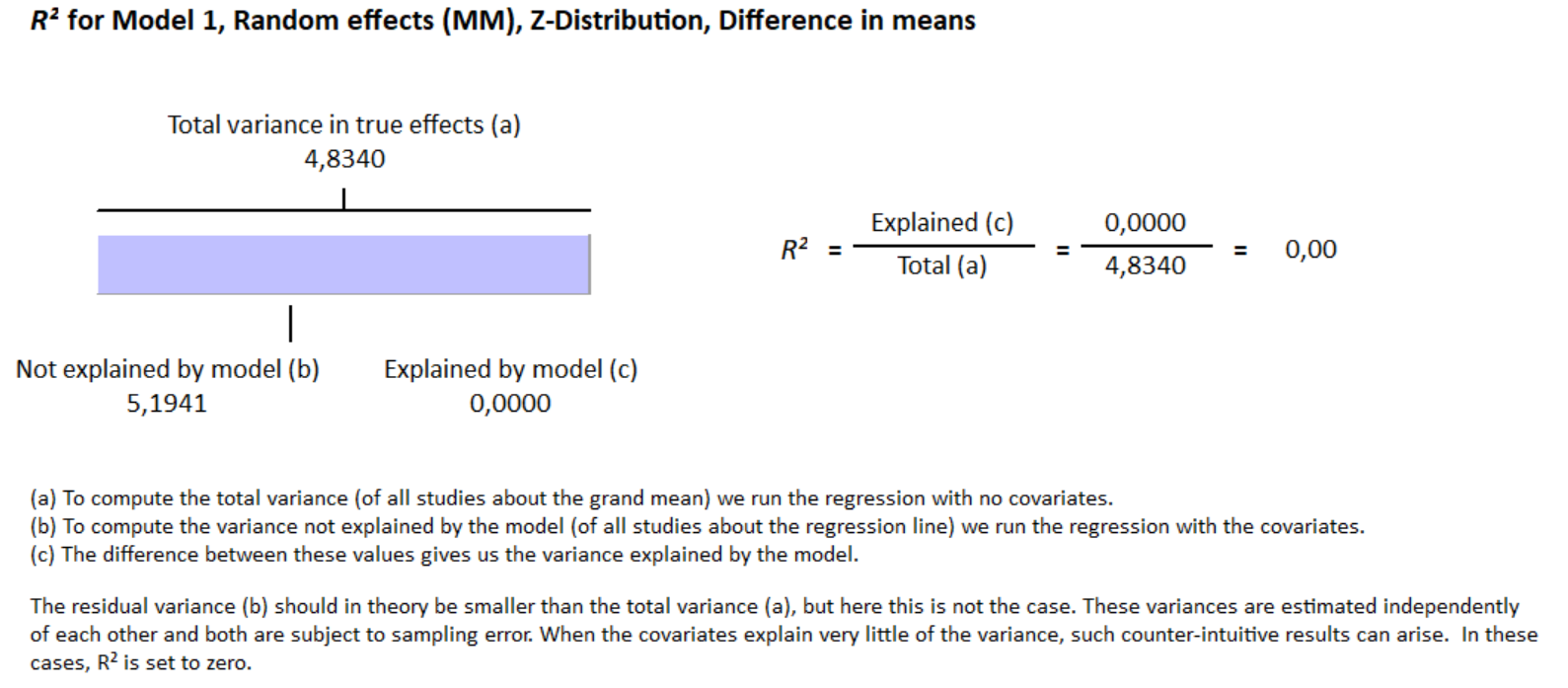


**S13 Fig** Meta-regression result for proportion of alcoholic etiology covariate have no influence on GLS reduction in group with cirrhosis with R^2^ = 0%
