# Supplementary material for: Evaluation of subclinical ventricular systolic dysfunction assessed using global longitudinal strain in liver cirrhosis: A systematic review, meta-analysis, and meta-regression
Source: PLoS One. 2022 Jun 7;17(6):e0269691. doi: 10.1371/journal.pone.0269691 (PMC9173645; doi:10.1371/journal.pone.0269691)
Supplement: S1 Table — (DOCX) [file pone.0269691.s018.docx]

**S1 Table.** Search Strategy Completed Using MeSH Terms and [All Fields]

| Database | Search Terms | Number of Studies Identified |
| --- | --- | --- |
| Cochrane Library | ("ventricular function" OR "ventricular dysfunction" OR myocardial):ti,ab,kw AND (echocardiography OR Ultrasonography OR echocardiographic OR "cardiac magnetic resonance" OR Speckle-tracking OR "Longitudinal Strain" OR "Tissue-Doppler"):ti,ab,kw AND ("liver cirrhosis" OR "End stage liver disease" OR cirrhotic):ti,ab,kw | 12 |
| EBSCOhost | ( TI "ventricular function" OR TI "ventricular dysfunction" OR TI myocardial OR AB "ventricular function" OR AB "ventricular dysfunction" OR AB myocardial) AND ( TI echocardiography OR TI Ultrasonography OR echocardiographic OR TI "cardiac magnetic resonance" OR TI Speckle-tracking OR TI "Longitudinal Strain" OR TI "Tissue-Doppler" OR AB echocardiography OR AB Ultrasonography OR AB echocardiographic OR AB "cardiac magnetic resonance" OR AB Speckle-tracking OR AB "Longitudinal Strain" OR AB "Tissue-Doppler") AND ( TI "liver cirrhosis" OR TI "End stage liver disease" OR TI cirrhotic OR AB "liver cirrhosis" OR AB "end stage disease renal" OR AB cirrhotic) | 85 |
| Open Grey | ("ventricular function" OR "ventricular dysfunction" OR myocardial) AND (echocardiography OR Ultrasonography OR echocardiographic OR "cardiac magnetic resonance" OR Speckle-tracking OR "Longitudinal Strain" OR "Tissue-Doppler") AND ("liver cirrhosis" OR "End stage liver disease" OR cirrhotic) | 0 |
| ProQuest | (ti("ventricular function") OR ti("ventricular dysfunction") OR ti(myocardial) OR ab("ventricular function") OR ab("ventricular dysfunction") OR ab(myocardial)) AND (ti(echocardiography) OR ti(Ultrasonography) OR ti(echocardiographic) OR ti("cardiac magnetic resonance") OR ti(Speckle-tracking) OR ti("Longitudinal Strain") OR ti("Tissue-Doppler") OR ab(echocardiography) OR ab(Ultrasonography) OR ab(echocardiographic) OR ab("cardiac magnetic resonance") OR ab(Speckle-tracking) OR ab("Longitudinal Strain") OR ab("Tissue-Doppler")) AND (ti("liver cirrhosis") OR ti("End stage liver disease") OR ti(cirrhotic) OR ab("liver cirrhosis") OR ab("end stage disease renal") OR ab(cirrhotic)) | 16 |
| Pubmed | ("ventricular function"[MeSH Terms] OR "ventricular dysfunction"[MeSH Terms] OR "ventricular function"[All Fields] OR "ventricular dysfunction"[All Fields] OR "myocardial"[All Fields]) AND ("echocardiography"[MeSH Terms] OR "Ultrasonography"[MeSH Terms] OR "echocardiography"[All Fields] OR "echocardiographic"[All Fields] OR "Ultrasonography"[All Fields] OR "cardiac magnetic resonance"[All Fields] OR "Speckle-tracking"[All Fields] OR "Longitudinal Strain" OR "Tissue-Doppler") AND ("liver cirrhosis"[MeSH Terms] OR "End stage liver disease"[MeSH Terms] OR "cirrhosis"[All Fields] OR "cirrhotic"[All Fields] OR "End stage liver disease"[All Fields]) | 260 |
| Pubmed Central | ("ventricular function"[MeSH Terms] OR "ventricular dysfunction"[MeSH Terms] OR "ventricular function"[All Fields] OR "ventricular dysfunction"[All Fields] OR "myocardial"[All Fields]) AND ("echocardiography"[MeSH Terms] OR "Ultrasonography"[MeSH Terms] OR "echocardiography"[All Fields] OR "echocardiographic"[All Fields] OR "Ultrasonography"[All Fields] OR "cardiac magnetic resonance"[All Fields] OR "Speckle-tracking"[All Fields] OR "Longitudinal Strain" OR "Tissue-Doppler") AND ("liver cirrhosis"[MeSH Terms] OR "End stage liver disease"[MeSH Terms] OR "cirrhosis"[All Fields] OR "cirrhotic"[All Fields] OR "End stage liver disease"[All Fields]) | 4341 |
| ScienceDirect | ("ventricular function") AND (echocardiography OR "cardiac magnetic resonance" OR "Speckle-tracking" OR "Longitudinal Strain" OR "Tissue-Doppler") AND ("liver cirrhosis" OR "End stage liver disease") | 633 |
| Total articles retrieved | | 5347 |
