# Supplementary material for: Evaluation of subclinical ventricular systolic dysfunction assessed using global longitudinal strain in liver cirrhosis: A systematic review, meta-analysis, and meta-regression
Source: PLoS One. 2022 Jun 7;17(6):e0269691. doi: 10.1371/journal.pone.0269691 (PMC9173645; doi:10.1371/journal.pone.0269691)
Supplement: S2 Table — (DOCX) [file pone.0269691.s019.docx]

**S2 Table.** Complete Characteristic of Included Study with Left and Right Ventricular Global Longitudinal Strain Results

| **Author (Year)** | **Country of Study Origin** | **Subjects (LC [%male] vs non-LC [%male])** | **Baseline Ages (LC vs non-LC, years old) [Median, Q1-Q3 / Mean ± SD]** | **Cirrhosis Etiology (n,%)** | **Cirrhosis Prognostic Score and Severity** | **Cirrhosis group comorbidity (n,%)** | **Control group comorbidity (n,%)** | **Ongoing Medical Therapy (n,%)** | **Exclusion Criteria** | **Diagnostic Test for GLS** | **Ejection Fraction (LC vs non-LC,%) and NT-ProBNP (LC, pg/ml) (Mean ± SD, Median, Q1-Q3)** | **Left Ventricular Longitudinal Strain (LC vs non-LC, %) (Median, Q1-Q3 / Mean ± SD)** | **Right Ventricular Longitudinal Strain (LC vs non-LC, %) [Median, Q1-Q3 / Mean ± SD]** | **Other Parameter (TAPSE [mm], RVFAC [%]) (LC vs non-LC)** |
| --- | --- | --- | --- | --- | --- | --- | --- | --- | --- | --- | --- | --- | --- | --- |
| CROSS SECTIONAL | | | | | | | | | | | | | | |
| Hammami R (2017)^45^ | Tunisia | 80 [52.5] vs 80 [N.R] | 55 ± 14 vs 51 ± 12 (p>0.05) | Viral (Hepatitis B and C): 42 (52.6%)  Cryptogenic: 21 (26.1)  Other causes: 17 (21.25) | Mean of MELD Score: 14.2 ± 4.98  Mean of CP Score: N.R  CP classification (n,%):  A: 24 (30)  B: 36 (45)  C: 20 (25) | Left ventricular hypertrophy: 39 (48.75) | Healthy | β-blocker: 62 (77.5) | Diabetes, hypertension, tabagism, pulmonary diseases, suspected coronary disease, age > 75 years or < 20 years, hepatocellular carcinoma, renal disease, anemia (hemoglobin <10 g/dl), thyroid disease, alcoholic cirrhosis, and abnormal electrocardiogram (repolarization disorders and rhythm anomalies) | 2D-STE (EchoPAC System) | EF: 60.48 ± 7.7 vs 62.2 ± 7.01 (p=0.08)  NT-ProBNP: N.R | Apical 4,2,3-chamber view: -19.8 ± 2.8 vs -22.01 ± 2.6 (p<0.001) | N.R | N.R |
| Rimbaş RC (2017)^15^ | Romania | 46 (65.2) vs 46 (72.5) | 57 ± 9 vs 55 ± 10 (p>0.05) | Alcoholic = 52%  Viral = 41%  Primary biliary cirrhosis = 4%  Cryptogenic = 2% | Mean of MELD Score:  13 ± 5  Mean of CP Score: 7 ± 2  CP classification (n,%):  A: 23 (50)  B: 16 (35)  C: 7 (15) | Diastolic dysfunction: 22 (47.8) | Healthy | N.R | history of any cardiac disease, have more than mild valvular heart disease, diabetes mellitus, ongoing pulmonary or renal disease, any disease or neoplasia with an estimated survival <12 month, hemochromatosis, encephalopathy greater than grade 2, uncontrolled ascites, recent gastrointestinal bleeding (<2 month) or ongoing infection and abnormal electrocardiogram | 2D-STE (EchoPAC System) | EF: 61 ± 8 vs 61 ± 9 (p>0.05)  NT-ProBNP: 251 ± 281 | Apical 4,2,3-chamber and short-axis view at level of papillary muscle:  -20.9 ± 3 vs -20.7 ± 2.8 (p>0.05) | Apical 4-chamber view:  -24 ± 5 vs -23 ± 4 (p>0.05) | TAPSE: 26 ± 5 vs 25 ± 3 (p>0.05)  RVFAC: 43 ± 12 vs 44 ± 8 (p>0.05) |
| Novo G (2018)^46^ | Italy | 39 [41.02] vs 39 [43.58] | 60 (48‐70) vs 59 (48‐67) (p>0.05) | Hepatitis C: 39 (100) | Median of MELD score: 7 (6-8)  Median of CP score: N.R  CP classification (n,%):  A: 39 (100) | Hypertension: 17 (43.58)  Diabete mellitus: 11 (28.20)  Dyslipidemia: 2 (5.13) | Healthy | N.R | Poor echocardiographic window, an EF < 50%, history of cardiac disease such as heart failure, arrhythmias**,** coronary artery disease, valve disease more than mild, cardiomyopathy and pace‐maker implantation, alcoholic liver disease or alcohol abuse, drug addiction, terminal comorbidities such as malignancy, end‐stage kidney disease and anaemia with haemoglobin less than 10 mg/dL. | 2D-STE (Velocity Vector Imaging) | EF: 58 (55‐60) vs 60 (55‐65) (p=0.786)  NT-ProBNP: N.R | Apical 4,2,3-chamber view:  Median: -18.1 (‐20.5 – -16.3) vs -21.2 (‐22.3 – -20.4) (p=0.001)  Mean estimation (Wan’s method): -18.3 ± 3.23 vs -21.3 ± 1.46 (p<0.001) | N.R | TAPSE: 22 (21‐24) vs 23 (20‐25) (p=0.702)  Mean Estimation (Wan’s method): 22.33 ± 2.31 vs 22.67 ± 3.85 (p>0.05) |
| Zamirian M (2019)^11^ | Iran | 20 [50] vs 10 [80] | 42.2 ± 4.7 vs 41.6 ± 4.7 (p>0.05) | N.R | Mean of MELD score: N.R  Mean of CP score: N.R  CP classification (n,%):  C: 20 (100) | Diastolic dysfunction: 4 (20) | Diastolic dysfunction: 1 (10) | N.R | History of coronary artery disease, clinical and preclinical symptoms or evidence of coronary artery disease, underlying arrhythmias or history of arrhythmia, severe valvular disease, congenital heart diseases, EF below 55%, diabetes. | 2D-STE (EchoPAC System) | EF: All group have > 55%  NT-ProBNP: N.R | Apical 4,2,3-chamber view:  -22.6 ± 2.4 vs -19.2 ± 1.9 (p=0.001) | N.R | N.R |
| Zhang K (2019)^47^ | Germany | 67 [44] vs 36 [49] | 53 ± 12 vs 47 ± 13 (p>0.05) | Alcoholic: 67 (100) | Mean of MELD score: 13.5 ± 8.9  Mean of CP score: N.R  CP classification (n,%): N.R | N.R | Healthy | N.R | Heart failure, coronary artery disease, atrial fibrillation, congenital heart disease, more than mild valvular heart disease, neoplastic disease, and renal failure, inadequate echocardiographic images | 2D-STE (EchoPAC System) | EF: All group have > 50%  NT-ProBNP: N.R | N.R | Apical 4- chamber view:  -19.8 ± 4.2 vs -21.8 ± 1.7 (p=0.005) | TAPSE: 25 ± 5 vs 25 ± 4 (p=0.809)  RVFAC: 48 ± 10 vs 47 ± 8 (p=0.483) |
| von Köckritz F (2021)^48^ | Germany | 80 [58.8] vs 30 [46.7] | 52.47 ± 10.24 vs 48.57 ± 12.93 (p=0.145) | Alcoholic: 31.25%  Hepatitis C: 12.5% Autoimmune: 10% NASH: 10%  PSC: 8.75%, Idiopathic: 8.75%  Other cause (cystic liver, Wilson’s disease, bile duct carcinoma, and Caroli syndrome): 18.75% | Mean of MELD score: 17 ± 6.65  Mean of CP score: N.R  CP classification (n,%):  C: 80 (100) | Diastolic dysfunction: 14 (17.5) | Healthy | N.R | History of coronary disease, heart failure, congenital heart disease, atrial fibrillation, or moderate-to-severe valvular disease | 2D-STE (EchoPAC system) | EF: 60.00 ± 5.17 vs 60.90 ± 4.70 (p=0.274)  NT-ProBNP: N.R | Apical 4,2,3-chamber view:  -21.39 ± 4.06 vs -18.73 ± 2.95 (p<0.001) | N.R | N.R |
| CASE CONTROL | | | | | | | | | | | | | | |
| Sampaio F (2013)^49^ | Portugal | 109 [78.9] vs 18 [17.2] | 54 (48–64) vs 51 (49–58) (p>0.05) | Alcoholic: 73 (67)  Viral: 27 (24.8)  Other: 9 (8.2) | Median of MELD score: 14 (10–18)  Median of CP Score: N.R  CP classification (n,%):  A: 37 (33.9)  B: 27 (24.8)  C: 45 (41.3) | Diastolic dysfunctoin: 44 (40.3) | Healthy | N.R | History of hypertension, cardiac disease, more than mild valvular heart disease or relevant ECG abnormalities. | 2D-STE (Velocity Vector Imaging) | EF: 64 (59–67) vs 61 (60–65) (p=0.42)  NT-ProBNP: 200 (90–555) [with diastolic dysfunction]; 177 (61–344) [without diastolic dysfunction] | Apical 4,2-chamber view:  -19.99% (-21.88 – -18.71) vs -22.02% (-23.10 – -21.18) (p=0.003)  Mean estimation (Wan’s method): -20.19 ± 2.38 vs -22.10 ± 1.54 (p<0.05) | N.R | TAPSE: 25.4 mm (22.0–28.2) vs 23.1 (21.5–26.2) (p=0.11)  Mean Estimation (Wan’s method): 25.2 ± 4.66 vs 23.6 ± 3.78 (p>0.05) |
| Al-Hwary S (2015)^50^ | Egypt | 20 [N.R] vs 40 [N.R] | 46.45 ± 6.29 vs 43.25 ± 5.11 (p>0.05) | N.R | Mean of MELD score: N.R  Mean of CP score: N.R  CP classification (n,%): All patients are stable cirrhotic patients | Hypertension | Healthy | N.R | N.R | 2D-STE (EchoPAC System) | EF: 67.47 ± 7.66 vs 67.79 ± 5.15 (p>0.05)  NT-ProBNP: N.R | Apical 4,2,3-chamber view:  -19.98 ± 7.65 vs -29.50 ± 5.92 (p<0.05) | N.R | N.R |
| Sampaio F (2015)^10^ | Portugal | 36 [83.3] vs 8 [62.5] | 54 (48-61) vs 52 (45-54) (p>0.05) | Alcoholic: 21 (58.3)  Viral: 10 (27.8)  Other causes: 5 (13.9) | Median of MELD score: 9 (7-11)  Median of CP Score: 5 (5-7)  CP classification (n,%)  A: 27 (75)  B: 8 (17.8)  C: 1 (7.2) | N.R | Referral for atypical chest pain | Diuretic: 7 (19.4) | History of hypertension, diabetes, cardiac disease or relevant ECG abnormalities, large volume ascites and/or unable to tolerate breath-holding, renal insufficiency (creatinine clearance ≤ 60 ml/min/1.73 m2) | FT-CMR | EF: 67 (64-72) vs 66.0 (64-70) (p=0.66)  NT-ProBNP: 58 (30-140) | Apical 4,2,3-chamber view:  Median: −18.9 (-16.0 − -20.5) vs -19.0 (-16.1 − -20.6) (p=0.96)  Mean estimation (Wan’s method): -18.47 ± 3.47 vs -18.57 ± 4.02 (p=0.94) | N.R | N.R |
| Anish PG (2019)^51^ | India | 55 [83.63] vs 30 [83.33] | 46.38 vs 45.56 (p>0.05) | N.R | Mean of MELD score: 12  MELD score > 12 (n,%): 22 (40)  MELD score < 12 (n,%): 33 (60)  Mean of CP score: N.R  CP classification (n,%): N.R | Pulmonary artery hypertension: 18 (32.7)  Left ventricular hypertrophy: 26 (47.3) | Healthy | N.R | Ischemic and valvular heart disease, hypertension, primary myocardial disease, diabetes mellitus, uremia, severe anemia, patients with suboptimal echocardiographic images. | 2D-STE (EchoPAC System) | EF: 64.309±5.1 vs 63.5±3.9 (p=0.451)  NT-ProBNP: N.R | Apical 4,2,3-chamber view:  -19.52 ± 2.41 vs -23.66 ± 2.31 (p<0.0001) | N.R | N.R |
| Isaak A (2020)^52^ | Germany | 42 [55] vs 18 [72] | 57 ± 11 vs 54 ± 19 (p>0.05) | Alcoholic: 24 (57)  Viral hepatitis: 4 (12)  Autoimmune hepatitis: 3 (7)  NASH: 3 (7)  Hemochromatosis: 1 (2)  Congenital anomaly: 1 (2)  Cryptogenic: 5 (12) | Mean of MELD score:  CP A: 10 ± 2  CP B: 12 ± 5  CP C: 15 ± 5  Mean of CP score: N.R  CP classification (n,%)  A: 11 (26)  B: 20 (48)  C: 11 (26) | N.R | Healthy  volunteers, outpatients referred for nonspecific cardiac  symptoms, or exclusion of structural  heart disease | N.R | Advanced chronic kidney disease, contraindication for MRI, preexisting cardiac disease, malignant disease, acute clinical deterioration, refusal to participate in the study | FT-CMR | EF: 63 ± 8 vs 61 ± 4 (p=0.15)  NT-ProBNP: N.R | Apical 4,2-chamber and parasternal short axis views:  -18.5 ± 4.0 ± -22.5 ± 3.6 (p<0.001) | N.R | N.R |
| Koç DÖ (2020)^53^ | Turkey | 50 [62] vs 33 [51.5] | 57 ± 13 vs 55 ± 12 (p>0.05) | Viral Hepatitis: 30 (60)  NAFLD: 12 (24)  Alcoholic: 4 (8)  Other cause: 4 (8) | Mean of MELD score:  15.84 ± 7.92  MELD score > 15 (n,%): 25 (50)  MELD score < 15 (n,%): 25 (50)  Mean of CP score: N.R  CP classification (n ,%)  A: 19 (38)  B: 14 (28)  C: 17 (34) | N.R | Healthy | N.R | History of hypertension, cardiac disease, relevant electrocardiogram abnormalities, malignancy, or active infection | 2D-STE (EchoPAC system) | EF: 55.94 ± 9.65 vs 73.52 ± 5.26 (p=0.001)  NT-ProBNP: N.R | Apical 4,2-chamber view:  -19.42 ± 2.83 vs -19.49 ± 2.33 (p>0.05) | Basal, middle, apical segment and ventricular septum view:  17.05 ± 3.49 vs 22.61 ± 0.93 (p=0.001) | N.R |
| PROSPECTIVE COHORT | | | | | | | | | | | | | | |
| Altekin RE (2014)^54^ | Turkey | 38 [63.2] vs 37 [54.1] | 48.3 ± 12.4 vs 45.4 ± 8.6 (p>0.05) | Viral (Hepatitis B and C): 23 (60.5)  Cryptogenic: 10 (26.3)  Biliary: 5 (13.1) | Mean of MELD score: 11.76 ± 4.92  Mean of CP score: N.R  CP classification (n,%):  A: 23 (60.5)  B: 12 (31.6)  C: 3 (7.9) | N.R | Healthy | N.R | Coronary artery disease, severe or moderate heart valve disease, diabetes mellitus, hypertension, New York Heart Association class III-IV heart failure, pericarditis or massive pericardial effusion, cardiac rhythm anomalies, low ejection fraction (EF<60%), suboptimal echocardiographic images (especially those with poor image quality for the 2D-STE analysis and with obscured endocardial borders on the images), body mass index (BMI) >35 kg/m2, any metabolic or systemic diseases other than liver disease that might disrupt cardiac structure or function, smoking, and previous liver transplants that ended with rejection. | 2D-STE (EchoPAC System) | EF: 70.26 ± 4 vs 66.16 ± 4.09 (p=0.033)  NT-ProBNP: N.R | Apical 4,2,3-chamber and parasternal short axis view:  -20.57 ± 2.1 vs -28.74 ± 3.11 (p<0.001) | N.R | N.R |
| Huang CH (2019)^36^ | Taiwan | 80 [80] vs 29 [65.5] | 48.5 (45.0–59.0) vs 49.0 (43.0–52.5) (p>0.05) | Alcoholic: 28 (25.7)  Hepatitis B: 22 (20.2)  Hepatitis C: 30 (27.5) | Mean of MELD score:  Liver cirrhosis with CCM (n=22): 15.9 ± 8.3  Liver cirrhosis without CCM (n=57): 15.3 ± 7.9  Mean of CP score: N.R  CP classification (n,%):  A: 31 (38.75)  B/C: 49 (61.25) | Diastolic dysfunction: 27 (34.2) | Healthy | N.R | Diabetes mellitus, hypertension or hypotension at enrollment, shock, ESRD (end-stage renal disease, heart disease, severe alcoholic hepatitis, acute liver failure. | 2D-STE (EchoPAC System) | EF: 70.0±7.3 vs 69.1±7.1 (p=0.572)  NT-ProBNP: N.R | Apical 4,2,3-chamber view:  Median: -21.5 (-22.4 – -20.4) vs -20.2 (-23.0 – -19.1) (p=0.108)  Mean estimation (Wan’s method): -21.43 ± 1.51 vs -20.77 ± 3.04 (p=0.136) | N.R | N.R |
| İnci SD (2019)^55^ | Turkey | 40 [70] vs 26 [61.54] | 46.2 ± 10.1 vs 42.2 ± 8.6 (p>0.05) | N.R | Mean of MELD score: N.R  Mean of CP score: N.R  CP classification (n,%):  C: 40 (100) | N.R | Healthy | N.R | Coronary artery disease, systolic dysfunction, diabetes mellitus, hypertension, a cardiac rhythm other than sinus, valvular heart heart valve surgery, hypertrophic cardiomyopathy, renal dysfunction, chronic obstructive pulmonary  disease, hepatopulmonary syndrome and pulmonary hypertension, sepsis, peripheral artery arterial disease. | 2D-STE (Velocity Vector Imaging) | EF: 59.7±2.7 vs 60.5±2.8 (p=0.002)  NT-ProBNP: N.R | Apical 4-chamber view: -16.0 ± 3.2 vs -17.6 ± 2.2 (p=0.003)  Apical 2-chamber: -16.2 ± 3.3 vs -18.7 ± 2.1 (p=0.002) | Apical 4- chamber view:  -19.2 ± 3.5 vs -21.5 ± 3.6 (p=0.003) | N.R |
| Özdemir E (2019)^56^ | Turkey | 40 [33] vs 40 [33] | 42.8 ± 8.8 vs 42.5 ± 11.4 (p>0.05) | Hepatitis B = 40 (100) | Mean of MELD score: N.R  Mean of CP score: N.R  CP classification (n,%): N.R | N.R | Healthy | N.R | Moderate to severe valve insufficiency or stenosis, cardiomyopathy, atrial fibrillation, diagnosed heart failure, diagnosed coronary artery disease, diabetes mellitus, arterial hypertension, congenital heart disease, pregnancy, lactation, chronic lung disease (diagnosed chronic obstructive pulmonary disease, asthma, interstitial lung disease etc.), hepatic dysfunction (abnormal ALT, AST, GGT, ALP, bilirubin, and INR values) or renal dysfunction (if serum creatinine value is outside of the normal laboratory values for the age), thyroid dysfunction (if TSH, free T4 and free T3 are outside of the normal laboratory values for the age), or anemia (hemoglobin values < 14 g per deciliter for men and <12 g per deciliter for women), HIV, hepatitis C virus co-infection | 2D-STE (Automated Cardiac Motion  Quantification Stress Echo - QLAB System) | EF: 61.7 ± 8.2 vs 62.6 ± 5.8 (p=0.583)  NT-ProBNP: N.R | Apical 4,2,3-chamber and parasternal short axis view:  -19.9 ± 3.4 vs -22.8 ± 1.9 (p<0.001) | N.R | N.R |
| Kim HM (2020)^57^ | South Korea | 33 [75.8] vs 17 [55] | 56.3 ± 9.9 vs 65.0 ± 14.8 (p>0.05) | Viral (Hepatitis B and C): 20 (60.6%)  Alcoholic: 9 (27.3)  Autoimmune hepatitis: 2 (6.1)  Cryptogenic: 2 (6.1) | Mean of MELD score: 18.8 ± 7.4  Mean of CP score: 9.8 ± 2.4  CP classification (n,%)  A/B: 10 (30.3)  C: 23 (69.7) | Hypertension: 8 (24.2)  Diabetes mellitus: 9 (27.3) | Healthy | Diuretic: 17 (51.5)  β-blocker: 8 (24.2)  ACE-I/ARB: 3 (9.1) | Aged < 18 years, decreased kidney function (estimated glomerular filtration rate < 30 mL/min/1.73m2), documented history of cardiovascular diseases including coronary  artery disease, and other forms of myocardial disease, and acute liver failure without cirrhosis. | 2D-STE (EchoPAC System) | EF: 66.0 ± 5.2 vs 62.7 ± 5.6 (p=0.049)  NT-ProBNP: N.R | Apical 4,2,3-chamber view:  -24.2 ± 2.7 vs -18.6 ± 2.2 (p<0.001) | N.R | N.R |
| Chen Y (2016)^14^ | China | 103 [74.8] vs 48 [66.7]  103 cirrhotic patients were classified into:  Undergoing LTx: 41  Without LTx: 26  Refusing Echo follow up: 14  Died during study period: 22 | 54.9 ± 7.3 vs 53.5 ± 7.9 (p>0.05) | *Undergoing LTx (n=41)*  Alcoholic: 6 (14.6)  Viral: 30 (73.2)  Others: 5  (12.2%)  *Without LTx (n=26)*  Alcohol: 3 (11.5)  Viral: 17 (65.4)  Others: 6  (23.1)  *Refusing Echo follow up:* N.R  *Died during study period:* N.R | *Undergoing LTx (n=41)*  Mean of MELD score: 21.3 ± 8.9  Mean of CP Score: N.R  CP classification (n,%):  A: 7 (17.1)  B: 11 (26.8)  C 23 (56.1)  *Without LTx (n=26)*  Mean of MELD score: 12.2 ± 5.6  Mean of CP Score: N.R  CP classification (n,%):  A: 12 (46.2)  B: 10 (38.5)  C: 4 (15.3)  *Refusing Echo follow up:* N.R  *Died during study period:* N.R | Hypertension: 26 (25.2%)  Diabetes mellitus: 21 (20.4) | Hypertension: 7 (14.5%)  Diabetes Mellitus: 5 (10.4%) | Diuretic: 25 (24.3)  β-blocker: 24 (23.3)  ACE-I/ARB: 5 (4.9)  CCB: 20 (19.4) | Documented history of cardiovascular disease including coronary artery disease, myocardial infarction, stroke or peripheral vascular disease, acute liver failure, LTx not related to liver cirrhosis, hepato-pulmonary syndrome and pulmonary hypertension | 2D-STE (EchoPAC System) | EF: 65.1 ± 4.8 vs 64.1 ± 4.4 (p=0.19)  NT-ProBNP: N.R | Apical 4,2,3-chamber view:  -18.6 ± 2.6 vs -20.1 ± 2.8 (p<0.01) | Apical 4-chamber view:  -21.2 ± 4.4 vs -23.0 ± 2.6 (p<0.01) | TAPSE: 23 ± 4 vs 23 ± 2 (p=0.77)  RVFAC: 53 ± 8 vs 55 ± 6 (p=0.06) |
| Hassan AAA (2019)^58^ | Egypt | 45 [42] vs 30 [53] | 47.13 ± 9.2 vs 46.8 ± 8.9 (p>0.05) | Hepatitis C: 45 (100) | Mean of MELD score: N.R  Mean of CP score: N.R  CP classification (n,%):  A: 15 (33)  B: 15 (33)  C: 15 (33) | N.R | Healthy | N.R | Hypertensive or diabetic patients, pre-existing cardiac disease like rheumatic heart disease, congenital heart disease, other pre-existing cardiovascular disease like myocarditis owing to infective etiology, heart muscle diseases like cardiomyopathies | 2D-STE (EchoPac System) | EF: 60.9 ±7.3 vs 59.6±3.8 (p=0.2)  NT-ProBNP: N.R | Apical 4,2,3-chamber view:  -19.5 ± 2.7 vs -20.7 ± 4.3 (p=0.04) | N.R | N.R |
| Ibrahim MG (2020)^59^ | Egypt | 50 [42] vs 50 [38] | 52 ± 12.04 vs 46.76 ± 12.1 (p>0.05) | Hepatitis C: 50 (100) | Mean of MELD score: N.R  Mean of CP score: N.R  CP classification (n,%):  A: 38 (76)  B: 12 (24) | Hypertension: 28 (56)  Diabetes mellitus: 14 (28)  Chronic Hepatitis C infection: 50 (100) | Hypertension: 13 (26)  Diabetes mellitus: 7 (14)  Chronic Hepatitis C infection: 50 (100) | N.R | History of arrhythmias or a current arrhythmia; any type of cardiomyopathy; coronary heart diseases; valvular heart disease; uncontrolled hypertension; previously treated chronic HCV patients; concomitant infection with either of hepatitis B virus, human immunodeficiency virus or bilharziasis; patients with an estimated glomerular filtration rate less than 30 ml/min; pregnant women; those with any form of autoimmune diseases; those with a history of malignancy | 2D-STE (EchoPAC System) | EF: 66.04 ± 2.98 vs 57.3 ± 3.71 (p<0.05)  NT-ProBNP: N.R | Apical 4,2,3-chamber view:  Median: -20 (-26 – -16.5) vs -20 (-28 – -17)  Mean estimation (Wan’s method): -20.83 ± 7.25 vs -21.67 ± 8.4 (p= 0.59) | Apical 4-chamber view:  Median: -22 (-30 to -17) vs -22 (-30 to -17)  Mean estimation (Wan’s method): -23 ± 9.92 vs -23 ± 9.92 (p=1.00) | TAPSE: 24.56 ± 3.08 vs 24.06 ± 2.65 (p>0.05)  RVFAC: 45.72 ± 4.88 vs 45.64 ± 4.89 (p>0.05) |

2D STE: 2 dimension speckle-tracking echocardiography; ACE-I: angiotensin converting enzyme inhibitor; ARB: angiotensin receptor blocker; CCB: calcium-channel blocker; EF: ejection fraction; FT-CMR: feature tracking cardiac magnetic resonance; LC: liver cirrhosis; LTx: liver transplantation; MELD: model for end-stage liver disease; NAFLD: non-alcoholic fatty liver disease; NASH: non-alcoholic steatohepatitis; N.R: not reported; NT-proBNP: N-terminal pro-brain natriuretic peptide; PSC: Primary sclerosing cholangitis; RVFAC: right ventricle fractional area change; TAPSE: tricuspid annular plane systolic excursion
