# Supplementary material for: Evaluation of subclinical ventricular systolic dysfunction assessed using global longitudinal strain in liver cirrhosis: A systematic review, meta-analysis, and meta-regression
Source: PLoS One. 2022 Jun 7;17(6):e0269691. doi: 10.1371/journal.pone.0269691 (PMC9173645; doi:10.1371/journal.pone.0269691)
Supplement: S3 Table — (DOCX) [file pone.0269691.s020.docx]

**S3 Table.** Newcastle Ottawa Scale for Cross-Sectional Studies

| Study | Selection | | | | | Comparability | | Outcome | | | | Overall Total |
| --- | --- | --- | --- | --- | --- | --- | --- | --- | --- | --- | --- | --- |
|  | Sample represents target population | Sample size adequate or justified | Non-respondents | Ascertainment of the exposure (risk factor) | Subtotal | The study control for the most important factor | The study control for any additional factor | Outcome assessment | Statistical test | Subtotal | | Total / 10 |
| Hammami R (2017) | 1 | 0 | 1 | 2 | 4 | 1 | 1 | 2 | 1 | 3 | 9 | |
| Rimbaş RC (2017) | 1 | 0 | 0 | 2 | 3 | 1 | 1 | 2 | 1 | 3 | 8 | |
| Novo G (2018) | 0 | 0 | 1 | 2 | 3 | 1 | 1 | 2 | 1 | 3 | 8 | |
| Zamirian M (2019) | 0 | 0 | 1 | 2 | 3 | 1 | 1 | 2 | 1 | 3 | 8 | |
| Zhang K (2019) | 0 | 0 | 1 | 2 | 3 | 1 | 1 | 2 | 1 | 3 | 8 | |
| von Köckritz F (2021) | 0 | 0 | 1 | 2 | 3 | 1 | 1 | 2 | 1 | 3 | 8 | |
