# Supplementary material for: Evaluation of subclinical ventricular systolic dysfunction assessed using global longitudinal strain in liver cirrhosis: A systematic review, meta-analysis, and meta-regression
Source: PLoS One. 2022 Jun 7;17(6):e0269691. doi: 10.1371/journal.pone.0269691 (PMC9173645; doi:10.1371/journal.pone.0269691)
Supplement: S4 Table — (DOCX) [file pone.0269691.s021.docx]

**S4 Table.** Newcastle Ottawa Scale for Case-Control Studies

| Study | Selection | | | | | Comparability | Exposure | | | | Overall Total |
| --- | --- | --- | --- | --- | --- | --- | --- | --- | --- | --- | --- |
|  | Case definition adequate or not | Representativeness of the cases | Selection of controls | Definition of controls | Subtotal |  | Ascertainment of exposure | Same method of ascertainment for cases and controls | Non-response rate | Subtotal | Total / 9 |
| Sampaio F (2013) | 1 | 1 | 1 | 1 | 4 | 2 | 1 | 1 | 0 | 2 | 8 |
| Al-Hwary S (2015) | 0 | 1 | 1 | 1 | 3 | 2 | 1 | 1 | 0 | 2 | 7 |
| Sampaio F (2015) | 1 | 1 | 1 | 1 | 4 | 2 | 1 | 1 | 0 | 2 | 8 |
| Anish PG (2019) | 1 | 1 | 1 | 1 | 4 | 2 | 1 | 1 | 0 | 2 | 8 |
| Isaak A (2020) | 1 | 1 | 1 | 1 | 4 | 2 | 1 | 1 | 0 | 2 | 8 |
| Koç DÖ (2020) | 1 | 1 | 1 | 1 | 4 | 2 | 1 | 1 | 0 | 2 | 8 |
