# Supplementary material for: Evaluation of subclinical ventricular systolic dysfunction assessed using global longitudinal strain in liver cirrhosis: A systematic review, meta-analysis, and meta-regression
Source: PLoS One. 2022 Jun 7;17(6):e0269691. doi: 10.1371/journal.pone.0269691 (PMC9173645; doi:10.1371/journal.pone.0269691)
Supplement: S5 Table — (DOCX) [file pone.0269691.s022.docx]

**S5 Table.** Newcastle Ottawa Scale for Cohort Studies

| Study | Selection | | | | | Comparability | Exposure | | | | Overall Total |
| --- | --- | --- | --- | --- | --- | --- | --- | --- | --- | --- | --- |
|  | Representativeness of the exposed cohort | Selection of the non exposed cohort | Ascertainment of exposure | No presence of outcome | Subtotal |  | Ascertainment of outcome | Follow-up duration | Adequacy of follow up of cohorts | Subtotal | Total / 9 |
| Altekin RE (2014) | 0 | 0 | 1 | 1 | 2 | 2 | 1 | 0 | 1 | 2 | 6 |
| Huang CH (2019) | 1 | 1 | 1 | 1 | 4 | 2 | 1 | 1 | 1 | 3 | 9 |
| İnci SD (2019) | 0 | 1 | 1 | 1 | 3 | 2 | 1 | 0 | 1 | 2 | 7 |
| Özdemir E (2019) | 0 | 0 | 1 | 1 | 2 | 2 | 1 | 1 | 1 | 3 | 7 |
| Kim HM (2020) | 1 | 0 | 1 | 1 | 3 | 0 | 1 | 1 | 1 | 3 | 6 |
| Chen Y (2016) | 1 | 0 | 1 | 1 | 3 | 2 | 1 | 1 | 1 | 3 | 8 |
| Hassan AAA (2019) | 0 | 0 | 1 | 1 | 2 | 2 | 1 | 1 | 1 | 3 | 7 |
| Ibrahim MG (2020) | 0 | 1 | 1 | 1 | 3 | 1 | 1 | 1 | 1 | 3 | 8 |
