# Supplementary material for: Evaluation of subclinical ventricular systolic dysfunction assessed using global longitudinal strain in liver cirrhosis: A systematic review, meta-analysis, and meta-regression
Source: PLoS One. 2022 Jun 7;17(6):e0269691. doi: 10.1371/journal.pone.0269691 (PMC9173645; doi:10.1371/journal.pone.0269691)
Supplement: S6 Table — (DOCX) [file pone.0269691.s023.docx]

**S6 Table.** Sensitivity Analysis for Mean Difference of Left Ventricular Global Longitudinal Strain from Cirrhotic versus Non-Cirrhotic Patients

| **Omitted Study** | **Mean difference (95% CI)** | **Heterogeneity** | | | **P value** |
| --- | --- | --- | --- | --- | --- |
|  |  | **Tau^2^** | **Q** | **I^2^** |  |
| Sampaio F (2013) | -1.41 (-2.89 – 0.07) | 9.52 | 383.28 | 96% | P<0.00001 |
| **Altekin RE (2014)** | **-1.02 (-2.21 – 0.18)** | **5.93** | **259.29** | **93%** | **P<0.00001** |
| Sampaio F (2015) | -1.49 (-2.89 – -0.10) | 8.50 | 383.46 | 96% | P<0.00001 |
| Al-Hwary S (2015) | -1.08 (-2.44 – 0.28) | 8.03 | 364.14 | 95% | P<0.00001 |
| Chen Y (2016) | -1.43 (-2.90 – 0.04) | 9.35 | 384.29 | 96% | P<0.00001 |
| Hammami R (2017) | -1.39 (-2.87 – 0.09) | 9.50 | 381.15 | 96% | P<0.00001 |
| Rimbaş RC (2017) | -1.53 (-2.96 – -0.10) | 8.80 | 375.93 | 95% | P<0.00001 |
| Novo G (2018) | -1.34 (-2.78 – 0.09) | 8.90 | 376.79 | 95% | P<0.00001 |
| Anish PG (2019) | -1.27 (-2.68 – 0.13) | 8.51 | 357.66 | 95% | P<0.00001 |
| Özdemir E (2019) | -1.35 (-2.78 – 0.08) | 8.85 | 378.80 | 96% | P<0.00001 |
| Huang CH (2019) | -1.55 (-2.97 – -0.14) | 8.69 | 370.00 | 96% | P<0.00001 |
| Hassan AAA (2019) | -1.45 (-2.86 – -0.03) | 8.70 | 384.18 | 96% | P<0.00001 |
| İnci SD (2019) | -1.42 (-2.86 – 0.01) | 8.91 | 384.27 | 96% | P<0.00001 |
| Zamirian M (2019) | -1.70 (-3.05 – -0.35) | 7.85 | 346.08 | 95% | P<0.00001 |
| Isaak A (2020) | -1.29 (-2.70 – 0.11) | 8.49 | 378.49 | 96% | P<0.00001 |
| Ibrahim MG (2020) | -1.46 (-2.86 – -0.06) | 8.51 | 384.12 | 96% | P<0.00001 |
| Kim HM (2020) | -1.82 (-3.04 – -0.59) | 6.34 | 280.27 | 94% | P<0.00001 |
| Koç DÖ (2020) | -1.51 (-2.95 – -0.08) | 8.91 | 377.57 | 95% | P<0.00001 |
| von Köckritz F (2021) | -1.66 (-3.02 – -0.30) | 7.98 | 347.88 | 95% | P<0.00001 |
