# Supplementary material for: Evaluation of subclinical ventricular systolic dysfunction assessed using global longitudinal strain in liver cirrhosis: A systematic review, meta-analysis, and meta-regression
Source: PLoS One. 2022 Jun 7;17(6):e0269691. doi: 10.1371/journal.pone.0269691 (PMC9173645; doi:10.1371/journal.pone.0269691)
Supplement: S7 Table — (DOCX) [file pone.0269691.s024.docx]

**S7 Table.** Sensitivity Analysis for Mean Difference of Left Ventricular Global Longitudinal Strain from Cirrhotic versus Non-Cirrhotic Patients after Omission of Study by Altekin, et al.

| **Omitted Study** | **Mean difference (95% CI)** | **Heterogeneity** | | | **P value** |
| --- | --- | --- | --- | --- | --- |
|  |  | **Tau^2^** | **Q** | **I^2^** |  |
| Sampaio F (2013) | -0.97 (-2.26 – 0.33) | 6.68 | 255.31 | 94% | P<0.00001 |
| Sampaio F (2015) | -1.06 (-2.29 – 0.17) | 6.01 | 258.86 | 94% | P<0.00001 |
| Al-Hwary S (2015) | -0.66 (-1.83 – 0.51) | 5.46 | 237.07 | 93% | P<0.00001 |
| Chen Y (2016) | -0.99 (-2.29 – 0.30) | 6.63 | 258.51 | 94% | P<0.00001 |
| Hammami R (2017) | -0.95 (-2.24 – 0.34) | 6.60 | 251.56 | 94% | P<0.00001 |
| Rimbaş RC (2017) | -1.10 (-2.35 – 0.16) | 6.26 | 254.38 | 94% | P<0.00001 |
| Novo G (2018) | -0.89 (-2.14 – 0.35) | 6.13 | 247.21 | 94% | P<0.00001 |
| Anish PG (2019) | -0.81 (-2.01 – 0.38) | 5.57 | 223.82 | 93% | P<0.00001 |
| Özdemir E (2019) | -0.90 (-2.15 – 0.35) | 6.13 | 250.19 | 94% | P<0.00001 |
| Huang CH (2019) | -1.12 (-2.37 – 0.13) | 6.16 | 249.76 | 94% | P<0.00001 |
| Hassan AAA (2019) | -1.01 (-2.25 – 0.24) | 6.16 | 259.27 | 94% | P<0.00001 |
| İnci SD (2019) | -0.98 (-2.25 – 0.28) | 6.30 | 258.69 | 94% | P<0.00001 |
| Zamirian M (2019) | -1.27 (-2.44 – -0.10) | 5.38 | 226.99 | 93% | P<0.00001 |
| Isaak A (2020) | -0.85 (-2.07 – 0.37) | 5.90 | 251.48 | 94% | P<0.00001 |
| Ibrahim MG (2020) | -1.02 (-2.25 – 0.20) | 6.02 | 259.26 | 94% | P<0.00001 |
| **Kim HM (2020)** | **-1.39 (-2.40 – -0.37)** | **3.87** | **166.40** | **90%** | **P<0.00001** |
| Koç DÖ (2020) | -1.08 (-2.35 – 0.19) | 6.35 | 255.79 | 94% | P<0.00001 |
| von Köckritz F (2021) | -1.23 (-2.42 – -0.05) | 5.50 | 229.46 | 93% | P<0.00001 |
