# Supplementary material for: Evaluation of subclinical ventricular systolic dysfunction assessed using global longitudinal strain in liver cirrhosis: A systematic review, meta-analysis, and meta-regression
Source: PLoS One. 2022 Jun 7;17(6):e0269691. doi: 10.1371/journal.pone.0269691 (PMC9173645; doi:10.1371/journal.pone.0269691)
Supplement: S8 Table — (DOCX) [file pone.0269691.s025.docx]

**S8 Table.** Sensitivity Analysis for Mean Difference of Left Ventricular Global Longitudinal Strain from Cirrhotic versus Non-Cirrhotic Patients after Omission of Study by Altekin, et al. and Kim, et al.

| **Omitted Study** | **Mean difference (95% CI)** | **Heterogeneity** | | | **P value** |
| --- | --- | --- | --- | --- | --- |
|  |  | **Tau^2^** | **Q** | **I^2^** |  |
| Sampaio F (2013) | -1.36 (-2.48 – -0.24) | 4.44 | 164.89 | 91% | P<0.00001 |
| Sampaio F (2015) | -1.44 (-2.49 – -0.40) | 3.93 | 165.66 | 91% | P<0.00001 |
| Al-Hwary S (2015) | -1.07 (-2.05 – -0.09) | 3.40 | 145.80 | 90% | P<0.00001 |
| Chen Y (2016) | -1.39 (-2.50 – -0.28) | 4.38 | 166.36 | 91% | P<0.00001 |
| Hammami R (2017) | -1.34 (-2.45 – -0.23) | 4.38 | 162.39 | 91% | P<0.00001 |
| Rimbaş RC (2017) | -1.49 (-2.56 – -0.43) | 3.99 | 158.81 | 91% | P<0.00001 |
| Novo G (2018) | -1.28 (-2.34 – -0.21) | 4.01 | 157.95 | 91% | P<0.00001 |
| Anish PG (2019) | -1.18 (-2.19 – -0.18) | 3.48 | 137.75 | 89% | P<0.00001 |
| Özdemir E (2019) | -1.29 (-2.35 – -0.22) | 4.02 | 160.17 | 91% | P<0.00001 |
| Huang CH (2019) | -1.52 (-2.57 – -0.48) | 3.85 | 153.13 | 90% | P<0.00001 |
| Hassan AAA (2019) | -1.40 (-2.47 – -0.33) | 4.04 | 166.34 | 91% | P<0.00001 |
| İnci SD (2019) | -1.38 (-2.46 – -0.30) | 4.15 | 166.31 | 91% | P<0.00001 |
| Zamirian M (2019) | -1.67 (-2.62 – -0.72) | 3.07 | 129.38 | 88% | P<0.00001 |
| Isaak A (2020) | -1.23 (-2.28 – -0.19) | 3.84 | 160.18 | 91% | P<0.00001 |
| Ibrahim MG (2020) | -1.41 (-2.46 – -0.36) | 3.94 | 166.27 | 91% | P<0.00001 |
| Koç DÖ (2020) | -1.48 (-2.55 – -0.41) | 4.07 | 160.42 | 91% | P<0.00001 |
| von Köckritz F (2021) | -1.63 (-2.60 – -0.67) | 3.17 | 131.31 | 89% | P<0.00001 |
