# Supplementary material for: Evaluation of subclinical ventricular systolic dysfunction assessed using global longitudinal strain in liver cirrhosis: A systematic review, meta-analysis, and meta-regression
Source: PLoS One. 2022 Jun 7;17(6):e0269691. doi: 10.1371/journal.pone.0269691 (PMC9173645; doi:10.1371/journal.pone.0269691)
Supplement: S9 Table — (DOCX) [file pone.0269691.s026.docx]

**S9 Table.** Sensitivity Analysis for Mean Difference of Right Ventricular Global Longitudinal Strain from Cirrhotic versus Non-Cirrhotic Patients

| **Deleted Study** | **Mean difference (95% CI)** | **Heterogeneity** | | | **P value** |
| --- | --- | --- | --- | --- | --- |
|  |  | **Tau^2^** | **Q** | **I^2^** |  |
| Chen Y (2016) | -1.94 (-4.36 – 0.48) | 6.58 | 46.95 | 91% | P<0.00001 |
| Rimbaş RC (2017) | -2.58 (-4.42 – -0.74) | 3.57 | 33.28 | 88% | P<0.00001 |
| Zhang K (2019) | -1.89 (-4.33 – 0.55) | 6.72 | 48.40 | 92% | P<0.00001 |
| İnci SD (2019) | -1.85 (-4.11 – 0.41) | 5.72 | 50.14 | 92% | P<0.00001 |
| Ibrahim MG (2020) | -2.19 (-4.22 – -0.16) | 4.85 | 48.46 | 92% | P<0.00001 |
| **Koç DÖ (2020)** | **-1.30 (-2.43 – -0.18)** | **0.87** | **9.38** | **57%** | **P=0.05** |
