# Supplementary material for: Evaluation of subclinical ventricular systolic dysfunction assessed using global longitudinal strain in liver cirrhosis: A systematic review, meta-analysis, and meta-regression
Source: PLoS One. 2022 Jun 7;17(6):e0269691. doi: 10.1371/journal.pone.0269691 (PMC9173645; doi:10.1371/journal.pone.0269691)
Supplement: S10 Table — (DOCX) [file pone.0269691.s027.docx]

**S10 Table.** Sensitivity Analysis for Mean Difference of Right Ventricular Global Longitudinal Strain from Cirrhotic versus Non-Cirrhotic Patients after Omission of Study by Koç, et al.

| **Deleted Study** | **Mean difference (95% CI)** | **Heterogeneity** | | | **P value** |
| --- | --- | --- | --- | --- | --- |
|  |  | **Tau^2^** | **Q** | **I^2^** |  |
| Chen Y (2016) | -1.04 (-2.67 – 0.60) | 1.73 | 9.01 | 67% | P=0.03 |
| **Rimbaş RC (2017)** | **-1.90 (-2.62 – -1.18)** | **0.00** | **1.18** | **0%** | **P=0.76** |
| Zhang K (2019) | -0.99 (-2.55 – 0.58) | 1.52 | 8.37 | 64% | P=0.04 |
| İnci SD (2019) | -1.01 (-2.39 – 0.36) | 1.16 | 8.50 | 65% | P=0.04 |
| Ibrahim MG (2020) | -1.39 (-2.61 – -0.18) | 0.99 | 8.77 | 66% | P=0.03 |
