# Supplementary material for: Evaluation of subclinical ventricular systolic dysfunction assessed using global longitudinal strain in liver cirrhosis: A systematic review, meta-analysis, and meta-regression
Source: PLoS One. 2022 Jun 7;17(6):e0269691. doi: 10.1371/journal.pone.0269691 (PMC9173645; doi:10.1371/journal.pone.0269691)
Supplement: S11 Table — (DOCX) [file pone.0269691.s028.docx]

**S11 Table.** Sensitivity Analysis for Mean Difference of Right Ventricular Global Longitudinal Strain from Cirrhotic versus Non-Cirrhotic Patients after Omission of Study by Koç, et al. and Rimbaş, et al.

| **Deleted Study** | **Mean difference (95% CI)** | **Heterogeneity** | | | **P value** |
| --- | --- | --- | --- | --- | --- |
|  |  | **Tau^2^** | **Q** | **I^2^** |  |
| Chen Y (2016) | -1.97 (-2.90 – -1.04) | 0.00 | 1.13 | 0% | P=0.57 |
| Zhang K (2019) | -1.84 (-2.76 – -0.92) | 0.00 | 1.13 | 0% | P=0.57 |
| İnci SD (2019) | -1.82 (-2.61 – -1.03) | 0.00 | 0.94 | 0% | P=0.62 |
| Ibrahim MG (2020) | -1.97 (-2.70 – -1.24) | 0.00 | 0.23 | 0% | P=0.89 |
