# Supplementary material for: Evaluation of subclinical ventricular systolic dysfunction assessed using global longitudinal strain in liver cirrhosis: A systematic review, meta-analysis, and meta-regression
Source: PLoS One. 2022 Jun 7;17(6):e0269691. doi: 10.1371/journal.pone.0269691 (PMC9173645; doi:10.1371/journal.pone.0269691)
Supplement: S12 Table — (DOCX) [file pone.0269691.s029.docx]

**S12 Table.** Meta Regression Results and R^2^ for Study Design Covariate

| **Set** | **Covariate** | **Coefficient** | **Standard Error** | **95% Lower** | **95% Upper** | **Z-value** | **2-sided p value** | **Set** |
| --- | --- | --- | --- | --- | --- | --- | --- | --- |
|  | Intercept | -3,0646 | 1,3231 | -5,6579 | -0,4713 | -2,32 | 0,0205 |  |
| Study Design | Study Design: Cross Sectional | 3,2225 | 1,9247 | -0,5498 | 6,9948 | 1,67 | 0,0941 | Q=2,84, df=2, p=0,2419 |
| Study Design | Study Design: Prospective Cohort | 1,7984 | 1,7302 | -1,5928 | 5,1895 | 1,04 | 0,2986 | Q=2,84, df=2, p=0,2419 |
| **STATISTIC FOR THIS MODEL** | | | | | | | | |
| **Test of this model: Simultaneous test that all coefficients (excluding intercept) are zero** | | | | | | | | |
| Q = 2,84, df = 2, p = 0,2419 | | | | | | | | |
| **Goodness of fit: Test that unexplained variance is zero** | | | | | | | | |
| Tau² = 9,3153, Tau = 3,0521, I² = 95,43%, Q = 350,47, df = 16, p = 0,0000 | | | | | | | | |
| **COMPARISON OF THIS MODEL WITH THE NULL MODEL** | | | | | | | | |
| **Total between-study variance (intercept only)** | | | | | | | | |
| Tau² = 8,1867, Tau = 2,8612, I² = 95,07%, Q = 364,91, df = 18, p = 0,0000 | | | | | | | | |
| **Proportion of total between-study variance explained by this model** | | | | | | | | |
| R² analog = 0,00 (computed value is -0,14) | | | | | | | | |
