# Supplementary material for: Evaluation of subclinical ventricular systolic dysfunction assessed using global longitudinal strain in liver cirrhosis: A systematic review, meta-analysis, and meta-regression
Source: PLoS One. 2022 Jun 7;17(6):e0269691. doi: 10.1371/journal.pone.0269691 (PMC9173645; doi:10.1371/journal.pone.0269691)
Supplement: S13 Table — (DOCX) [file pone.0269691.s030.docx]

**S13 Table**. Meta Regression Results and R^2^ for Proportion of Male Subjects Covariate

| **Covariate** | **Coefficient** | **Standard Error** | **95% Lower** | **95% Upper** | **Z-value** | **2-sided p value** |
| --- | --- | --- | --- | --- | --- | --- |
| Intercept | -2,8339 | 3,5108 | -9,7149 | 4,0472 | -0,81 | 0,4196 |
| Male (%) | 0,0297 | 0,0559 | -0,0798 | 0,1392 | 0,53 | 0,5952 |
| **STATISTIC FOR THIS MODEL** | | | | | | |
| **Test of this model: Simultaneous test that all coefficients (excluding intercept) are zero** | | | | | | |
| Q = 0,28, df = 1, p = 0,5952 | | | | | | |
| **Goodness of fit: Test that unexplained variance is zero** | | | | | | |
| Tau² = 9,4817, Tau = 3,0792, I² = 95,54%, Q = 336,12, df = 15, p = 0,0000 | | | | | | |
| **COMPARISON OF THIS MODEL WITH THE NULL MODEL** | | | | | | |
| **Total between-study variance (intercept only)** | | | | | | |
| Tau² = 8,8836, Tau = 2,9805, I² = 95,35%, Q = 343,87, df = 16, p = 0,0000 | | | | | | |
| **Proportion of total between-study variance explained by this model** | | | | | | |
| R² analog = 0,00 (computed value is -0,07) | | | | | | |
