# Supplementary material for: Evaluation of subclinical ventricular systolic dysfunction assessed using global longitudinal strain in liver cirrhosis: A systematic review, meta-analysis, and meta-regression
Source: PLoS One. 2022 Jun 7;17(6):e0269691. doi: 10.1371/journal.pone.0269691 (PMC9173645; doi:10.1371/journal.pone.0269691)
Supplement: S15 Table — (DOCX) [file pone.0269691.s032.docx]

**S15 Table.** Meta Regression Results and R^2^ for MELD Score Covariate

| **Covariate** | **Coefficient** | **Standard Error** | **95% Lower** | **95% Upper** | **Z-value** |
| --- | --- | --- | --- | --- | --- |
| Intercept | -9,4251 | 3,1808 | -15,6594 | -3,1909 | -2,96 |
| Mean MELD Score | 0,5936 | 0,2249 | 0,1527 | 1,0345 | 2,64 |
| **STATISTIC FOR THIS MODEL** | | | | | |
| **Test of this model: Simultaneous test that all coefficients (excluding intercept) are zero** | | | | | |
| Q = 6,96, df = 1, p = 0,0083 | | | | | |
| **Goodness of fit: Test that unexplained variance is zero** | | | | | |
| Tau² = 6,3517, Tau = 2,5203, I² = 94,69%, Q = 207,08, df = 11, p = 0,0000 | | | | | |
| **COMPARISON OF THIS MODEL WITH THE NULL MODEL** | | | | | |
| **Total between-study variance (intercept only)** | | | | | |
| Tau² = 8,8034, Tau = 2,9671, I² = 96,14%, Q = 311,01, df = 12, p = 0,0000 | | | | | |
| **Proportion of total between-study variance explained by this model** | | | | | |
| R² analog = 0,28 | | | | | |
