# Supplementary material for: Evaluation of subclinical ventricular systolic dysfunction assessed using global longitudinal strain in liver cirrhosis: A systematic review, meta-analysis, and meta-regression
Source: PLoS One. 2022 Jun 7;17(6):e0269691. doi: 10.1371/journal.pone.0269691 (PMC9173645; doi:10.1371/journal.pone.0269691)
Supplement: S16 Table — (DOCX) [file pone.0269691.s033.docx]

**S16 Table.** Meta Regression Results and R^2^ for Proportion of Decompensated Cirrhosis Covariate

| **Covariate** | **Coefficient** | **Standard Error** | **95% Lower** | **95% Upper** | **Z-value** |
| --- | --- | --- | --- | --- | --- |
| Intercept | -4,8948 | 1,466 | -7,7681 | -2,0215 | -3,34 |
| Decompensated Cirrhosis (%) | 0,0583 | 0,0224 | 0,0145 | 0,1022 | 2,61 |
| **STATISTIC FOR THIS MODEL** | | | | | |
| **Test of this model: Simultaneous test that all coefficients (excluding intercept) are zero** | | | | | |
| Q = 6,81, df = 1, p = 0,0091 | | | | | |
| **Goodness of fit: Test that unexplained variance is zero** | | | | | |
| Tau² = 6,3542, Tau = 2,5207, I² = 92,94%, Q = 184,26, df = 13, p = 0,0000 | | | | | |
| **COMPARISON OF THIS MODEL WITH THE NULL MODEL** | | | | | |
| **Total between-study variance (intercept only)** | | | | | |
| Tau² = 7,8647, Tau = 2,8044, I² = 94,28%, Q = 244,58, df = 14, p = 0,0000 | | | | | |
| **Proportion of total between-study variance explained by this model** | | | | | |
| R² analog = 0,19 | | | | | |
