# Supplementary material for: Evaluation of subclinical ventricular systolic dysfunction assessed using global longitudinal strain in liver cirrhosis: A systematic review, meta-analysis, and meta-regression
Source: PLoS One. 2022 Jun 7;17(6):e0269691. doi: 10.1371/journal.pone.0269691 (PMC9173645; doi:10.1371/journal.pone.0269691)
Supplement: S17 Table — (DOCX) [file pone.0269691.s034.docx]

**S17 Table.** Meta Regression Results and R^2^ for Proportion of Alcholic Etiology Covariate

| **Covariate** | **Coefficient** | **Standard Error** | **95% Lower** | **95% Upper** | **Z-value** |
| --- | --- | --- | --- | --- | --- |
| Intercept | -0,6883 | 0,9911 | -2,6308 | 1,2543 | -0,69 |
| Alcoholic Etiology (%) | 0,0079 | 0,0281 | -0,0473 | 0,0631 | 0,28 |
| **STATISTIC FOR THIS MODEL** | | | | | |
| **Test of this model: Simultaneous test that all coefficients (excluding intercept) are zero** | | | | | |
| Q = 0,08, df = 1, p = 0,7786 | | | | | |
| **Goodness of fit: Test that unexplained variance is zero** | | | | | |
| Tau² = 5,1941, Tau = 2,2791, I² = 92,13%, Q = 139,86, df = 11, p = 0,0000 | | | | | |
| **COMPARISON OF THIS MODEL WITH THE NULL MODEL** | | | | | |
| **Total between-study variance (intercept only)** | | | | | |
| Tau² = 4,8340, Tau = 2,1986, I² = 91,69%, Q = 144,46, df = 12, p = 0,0000 | | | | | |
| **Proportion of total between-study variance explained by this model** | | | | | |
| R² analog = 0,00 (computed value is -0,07) | | | | | |
