# Supplementary material for: Evaluation of subclinical ventricular systolic dysfunction assessed using global longitudinal strain in liver cirrhosis: A systematic review, meta-analysis, and meta-regression
Source: PLoS One. 2022 Jun 7;17(6):e0269691. doi: 10.1371/journal.pone.0269691 (PMC9173645; doi:10.1371/journal.pone.0269691)
Supplement: S18 Table — (DOCX) [file pone.0269691.s035.docx]

**S18 Table.** Meta Regression Results and R^2^ for Proportion of Viral Etiology Covariate

| **Covariate** | **Coefficient** | **Standard Error** | **95% Lower** | **95% Upper** | **Z-value** |
| --- | --- | --- | --- | --- | --- |
| Intercept | 0,0064 | 1,7817 | -3,4857 | 3,4985 | 0 |
| Viral Etiology (%) | -0,0181 | 0,0269 | -0,0709 | 0,0347 | -0,67 |
| **STATISTIC FOR THIS MODEL** | | | | | |
| **Test of this model: Simultaneous test that all coefficients (excluding intercept) are zero** | | | | | |
| Q = 0,45, df = 1, p = 0,5008 | | | | | |
| **Goodness of fit: Test that unexplained variance is zero** | | | | | |
| Tau² = 9,1283, Tau = 3,0213, I² = 95,48%, Q = 265,20, df = 12, p = 0,0000 | | | | | |
| **COMPARISON OF THIS MODEL WITH THE NULL MODEL** | | | | | |
| **Total between-study variance (intercept only)** | | | | | |
| Tau² = 9,0792, Tau = 3,0132, I² = 95,46%, Q = 286,46, df = 13, p = 0,0000 | | | | | |
| **Proportion of total between-study variance explained by this model** | | | | | |
| R² analog = 0,00 (computed value is -0,01) | | | | | |
