# Supplementary material for: Evaluation of subclinical ventricular systolic dysfunction assessed using global longitudinal strain in liver cirrhosis: A systematic review, meta-analysis, and meta-regression
Source: PLoS One. 2022 Jun 7;17(6):e0269691. doi: 10.1371/journal.pone.0269691 (PMC9173645; doi:10.1371/journal.pone.0269691)
Supplement: S19 Table — (DOCX) [file pone.0269691.s036.docx]

**S19 Table.** Meta Regression Results and R^2^ for Baseline Left Ventricular Ejection Fraction in Cirrhotic Group Covariate

| **Covariate** | **Coefficient** | **Standard Error** | **95% Lower** | **95% Upper** | **Z-value** |
| --- | --- | --- | --- | --- | --- |
| Intercept | 9,4457 | 11,3854 | -12,8692 | 31,7607 | 0,83 |
| Mean LVEF in Cirrhotic (%) | -0,176 | 0,1797 | -0,5281 | 0,1761 | -0,98 |
| **STATISTIC FOR THIS MODEL** | | | | | |
| **Test of this model: Simultaneous test that all coefficients (excluding intercept) are zero** | | | | | |
| Q = 0,96, df = 1, p = 0,3273 | | | | | |
| **Goodness of fit: Test that unexplained variance is zero** | | | | | |
| Tau² = 8,3922, Tau = 2,8969, I² = 95,15%, Q = 329,82, df = 16, p = 0,0000 | | | | | |
| **COMPARISON OF THIS MODEL WITH THE NULL MODEL** | | | | | |
| **Total between-study variance (intercept only)** | | | | | |
| Tau² = 7,7044, Tau = 2,7757, I² = 94,89%, Q = 332,88, df = 17, p = 0,0000 | | | | | |
| **Proportion of total between-study variance explained by this model** | | | | | |
| R² analog = 0,00 (computed value is -0,09) | | | | | |
