# Supplementary material for: Evaluation of subclinical ventricular systolic dysfunction assessed using global longitudinal strain in liver cirrhosis: A systematic review, meta-analysis, and meta-regression
Source: PLoS One. 2022 Jun 7;17(6):e0269691. doi: 10.1371/journal.pone.0269691 (PMC9173645; doi:10.1371/journal.pone.0269691)
Supplement: S20 Table — (DOCX) [file pone.0269691.s037.docx]

**S20 Table.** Meta Regression Results and R^2^ for Newcastle Ottawa Scale Score Covariate

| **Covariate** | **Coefficient** | **Standard Error** | **95% Lower** | **95% Upper** | **Z-value** |
| --- | --- | --- | --- | --- | --- |
| Intercept | -6,8268 | 6,6738 | -19,9071 | 6,2535 | -1,02 |
| NOS Score | 0,7029 | 0,8627 | -0,9879 | 2,3937 | 0,81 |
| **STATISTIC FOR THIS MODEL** | | | | | |
| **Test of this model: Simultaneous test that all coefficients (excluding intercept) are zero** | | | | | |
| Q = 0,66, df = 1, p = 0,4152 | | | | | |
| **Goodness of fit: Test that unexplained variance is zero** | | | | | |
| Tau² = 8,4944, Tau = 2,9145, I² = 95,11%, Q = 347,94, df = 17, p = 0,0000 | | | | | |
| **COMPARISON OF THIS MODEL WITH THE NULL MODEL** | | | | | |
| **Total between-study variance (intercept only)** | | | | | |
| Tau² = 8,1867, Tau = 2,8612, I² = 95,07%, Q = 364,91, df = 18, p = 0,0000 | | | | | |
| **Proportion of total between-study variance explained by this model** | | | | | |
| R² analog = 0,00 (computed value is -0,04) | | | | | |
