# Supplementary material for: Evaluation of subclinical ventricular systolic dysfunction assessed using global longitudinal strain in liver cirrhosis: A systematic review, meta-analysis, and meta-regression
Source: PLoS One. 2022 Jun 7;17(6):e0269691. doi: 10.1371/journal.pone.0269691 (PMC9173645; doi:10.1371/journal.pone.0269691)
Supplement: Attachment — Submitted filename: REVISION NOTES PLOS ONE.docx [file pone.0269691.s039.docx]

**REVISION 1**

**Response from Academic Editor:**

**ACADEMIC EDITOR**: All issues raised by expert reviewers are required. Moreover, the authors should revise statistical approach in order to better support the conclusions.

**Response from Author:**

We have revise our statistical approach. The reporting of the trial sequential analysis has been improved by defining the mean difference a priori, before the trial sequential analysis run while also correcting the heterogeneity to reach a low heterogeneity (at least 25%). With this approach we report a more robust result. This was further described in the results section (Trial Sequential Analysis) and indicated in Figure S10 and Figure S11. This means that all result from meta analysis have shown a conclusive evidence with a low risk of a false positive result.

We calculated the information size required based on α = 5% significance level, and β = 20% (80% power) with an anticipated LV-GLS change of -1.43% and RV-GLS change of -1.95% in favor of cirrhotic groups. Heterogeneity was corrected to reach I2 of at least 25% (low heterogeneity). ***[PAGE 4]***

TSA result for LV-GLS and RV-GLS are shown in S10 Fig and S11 Fig, respectively. Both TSA showed that these studies cross trial monitoring boundary and confirm a lower absolute GLS in favor of cirrhotic group. This indicate that result of GLS in both ventricle have low risk of false positive results. After conducting TSA with sensitivity analysis that has been done previously in Fig 4 for LV-GLS and Figure S4 for RV-GLS, a further TSA is then generated for LV-GLS (S12 Fig) and RV-GLS (S13 Fig). From these TSA, the result is also conclusive and have low risk of false positive results. ***[PAGE 12]***

The conclusion have been better improved in accordance with the improved results by using a revised statistical approach

Despite the clinical and methodological heterogeneity, TSA revealed that current meta analysis have shown firm evidences that cirrhotic patients have a lower GLS when compared with controls. Future studies should provide a further analysis to elucidate whether there will be a significant difference of GLS in cirrhotic patients with different age, sex, cirrhosis severity and etiologies. ***[PAGE 16]***

**Response from Reviewer:**

Reviewer #1: Dear Authors, Thank you for the opportunity to read your work. After read it with attention the results seems to me sound. I'm not totally able to assess the statistical analysis, but it appear to me to be right. Please spent time to siimplify the text that is now too technical. I will read your revision version with pleasure.

**Response from Authors:**

We have simplify the text particularly the discussion part in order to be more readable. Several grammatical errors have also been corrected. ***[PAGE 2-16]***

**Response from Reviewer:**

Reviewer #2: The Authors performed a detailed metanalysis showing a significant reduction of LV-GLS and RV-GLS in cirrhotic patients. Overall, they performed an extensive search and analysis from as many as 5347 studies, yielding 20 studies (1738 pts; 2 studies with CMR, 18 with echo). Please find some comments:

-All patients presented by definition a LVEF >50%, but it is unknown how many patients presented cardiovascular comorbidities (hypertension ,diabetes, valvular heart disease, coronary artery disease, LV hypertrophy....) that might contribute to a decreased LV or RV GLS

**Response from Author:**

We have added additional sensitivity analysis related to cardiovascular comorbidities on its influence toward LV-GLS and RV-GLS result.

Moreover, in order to evaluate whether cardiovascular comorbidities could have contribution to a decreased LV-GLS, we excluded studies that include patients with hypertension, diabetes mellitus, and left ventricular hypertrophy. Further exclusion of three studies[14,44,57] with hypertension and diabetes mellitus patients showed similar result (MD:-1.55;95%CI,-2.37 – -0.73,p=0.0002; I2=73%,p=0.0003) while exclusion of a study[43] with left ventricular hypertrophy patients also showed simillar results (MD:-1.59;95%CI,-2.34 – -0.84,p<0.00001; I2=70%,p=0.0002). Other comorbidities such as coronary artery disease and valvular heart disease were excluded by all studies and therefore these variable did not influence current result. ***[PAGE 12]***

Moreover, in order to evaluate whether cardiovascular comorbidities could have contribution to a decreased RV-GLS, we excluded studies that include patients with hypertension, diabetes mellitus, and left ventricular hypertrophy. Further exclusion of two studies[14,57] with hypertension and diabetes mellitus patients showed similar result (MD:-2.09;95%CI,-3.05 – -1.13,p<0.0001; I2=0%,p=0.78). Among these studies, there were no study evaluating RV-GLS which include patients with left ventricular hypertrophy. Other comorbidities such as coronary artery disease and valvular heart disease were excluded by all studies and therefore these variable did not influence current result. ***[PAGE 13]***

**Response from Reviewer:**

-It is not clear which was the relationship between LVGLS and LV ejection fraction/MAPSE, or between RVGLS and TAPSE/FAC; did you analyse this point? Was there a difference between patients with bordeline LVEF and normal/high LVEF?

**Response from Author:**

We have added additional analysis comparing TAPSE and FAC between groups. Additional figure are added in suplementary files. However, there was no study reporting MAPSE and thus evaluation are limited.

**Other Parameters**

We found no significant difference of tricuspid annular plane systolic excursion (TAPSE) between cirrhotic versus non-cirrhotic patients based on all available data which was reported by 6 studies (MD:0.30;95%CI,-0.25 – 0.85, p=0.29; I2=0%,p=0.59) (S6 Fig).[14,15,44,45,47,57] Similarly, all 4 studies[14,15,45,57] reporting right ventricular fractional area change (RVFAC) between cirrhotic and non-cirrhotic patients showed no significant difference (MD:-0.56;95%CI,-1.85 – 0.73, p=0.40; I2=0%,p=0.44) (S7 Fig). ***[PAGE 14]***

**Response from Reviewer:**

-Besides an analysis on compensated/decompensated liver cirrhosis, were there differences according to ongoing medical therapy (diuretics? beta-blockers? ACE/ARB? MRA?) or to the presence of heart failure?

**Response from Author:**

We have added additional sensitivity analysis to evaluate the influence of heart failure (diastolic dysfunction, abnormal NT-proBNP result) and ongoing medical therapy that may affect the LV-GLS result. Moreover, we have update Table 1 by adding information regarding ongoing medical therapy and NT-proBNP. No study reporting the use of MRA in the population included and thus these variable was unable to be evaluated.

Additionaly, exclusion of two studies[15,47] which include patients with diastolic dysfunction revealed a similar result indicating a significant reduction of LV-GLS in cirrhotic patients (MD:-1.85;95%CI,-2.57 – -1.33,p<0.00001; I2=63%,p=0.003). In order to evaluate whether ongoing medical therapy (diuretics, beta-blockers, angiotensin converting enzyme inhibitor – ACE-I/angiotensin receptor blocker – ARB, calcium channel blocker – CCB) could influence LV-GLS, we exclude 3 studies[10,14,43] that report these medications in cirrhotic patients and the result showed that there is still a significant reduction of LV-GLS in cirrhotic patients (MD:-1.69;95%CI,-2.59 – -0.79,p=0.0002; I2=76%,p<0.0001). The effect of diuretic treatment that can be seen when a study[10] was excluded from the analysis also resulting in a similar result (MD:-1.72;95%CI,-2.40 – -1.04,p<0.00001; I2=71%,p=0.0002). Excluding a study[43] to evaluate the influence of beta-blocker treatment on the result also showing similar finding (MD:-1.59;95%CI,-2.34 – -0.84,p<0.0001; I2=70%,p=0.0002). ***[PAGE 12]***

The effect of diastolic dysfunction on RV-GLS results is unable to be evaluated because limited data availble on the remaining study. The effect of ongoing medical therapy (diuretics, beta-blockers, ACE-I/ARB, CCB) on RV-GLS result was evaluated by excluding a study[14] which still showing a significant reduction of RV-GLS in cirrhotic patients (MD:-1.97;95%CI,-2.90 – -1.04,p<0.0001; I2=0%,p=0.57). ***[PAGE 13]***
